# Understanding ACL injuries in volleyball: a systematic review of epidemiology and risk factors

**DOI:** 10.3389/fspor.2025.1675136

**Published:** 2025-12-08

**Authors:** Luana Beatriz Sassi, Ana Costa Miguel, Thiago Teixeira Serafim, Fábio Sprada de Menezes, Tamiris Beppler Martins, Rodrigo Okubo

**Affiliations:** 1 Department of Physiotherapy, State University of Santa Catarina, Florianópolis, Brazil; 2 Physical Therapy Graduate Program, State University of Santa Catarina, Florianópolis, Brazil; 3 Human Movement Sciences Graduate Program, State University of Santa Catarina, Florianópolis, Brazil

**Keywords:** anterior cruciate ligament injuries, volleyball, athletic injuries, sports, epidemiology

## Abstract

This systematic review aimed to comprehensively assess the epidemiological profile of anterior cruciate ligament (ACL) injuries among volleyball athletes, focusing on incidence, prevalence, injury mechanisms, and athlete characteristics such as sex, age, and court position. Following PRISMA-S guidelines, a thorough search was performed in PubMed, Web of Science, and SCOPUS, covering publications until December 26, 2024. Eligible studies included peer-reviewed cohort research reporting the incidence or prevalence of ACL injuries specifically in volleyball players. The methodological quality of these studies was critically evaluated using the adapted STROBE checklist. From an initial pool of 1,491 titles, 15 studies met the inclusion criteria, encompassing data from 3,313,248 athletes aged 10–70 years. The analysis revealed a substantial variation in ACL injury rates, largely influenced by factors such as exposure time, competition level, and injury mechanism. Notably, non-contact mechanisms were the most frequent cause of ACL injuries, predominantly occurring during spike landings in competitive settings. Female athletes, particularly high school players and those in the outside hitter position, exhibited the highest incidence of ACL injuries. These findings underscore the significant impact of athlete sex, playing level, and positional role on ACL injury risk in volleyball. The predominance of non-contact injuries highlights a critical need for sport-specific, targeted prevention strategies, especially among high-risk groups, such as adolescent female athletes and outside hitters. By identifying these key risk factors, this review provides a foundational understanding to inform tailored interventions, ultimately enhancing athlete safety and performance.

## 1 Introduction

Volleyball is widely recognized as a high-demand sport that, despite being non-contact in nature, involves intense biomechanical loading due to frequent jumping, landing, and multidirectional movements (1, 2). These actions, particularly during high-speed plays and spike landings, place significant stress on the lower limbs, making players vulnerable to musculoskeletal injuries, especially in the knee joint (1, 2). Among volleyball-related injuries, the anterior cruciate ligament (ACL) tear stands out due to its severity, long recovery period, and potential impact on athletic careers.

ACL injuries in volleyball typically occur via non-contact mechanisms, influenced by sex, age, competition level, and playing position (3–5). Female athletes, particularly adolescents, have a higher risk than their male peersdue to anatomical, hormonal, and neuromuscular differences (6). These injuries often result in prolonged rehabilitation and reduced rates of return to sport, with fewer than 65% of athletes regaining pre-injury performance levels (7), underscoring the relevance of volleyball as a model for injury-prevention research.

Although several studies have examined ACL injury rates in volleyball players, no previous systematic review has synthesized the available evidence focusing specifically on this population. Existing reviews across multiple sports or focused solely on female athletes offer limited applicability to volleyball because they merge heterogeneous injury mechanisms, exposure definitions, and positional demands (3, 8). Importantly, they do not address volleyball-specific aspects such as asymmetrical spike-landing biomechanics, positional movement patterns, or differences in exposure measurement, which restricts the ability to generate sport-specific prevention recommendations (9, 58).

Because the included studies reported either incidence or prevalence, both measures were incorporated to provide a more complete understanding of the ACL injury burden in volleyball. Therefore, the aim of this systematic review was to analyze the epidemiological profile of ACL injuries in volleyball athletes, including incidence, prevalence, injury mechanisms, and athlete characteristics such as sex, age category, and court position. This study, registered in PROSPERO and conducted in accordance with PRISMA-S guidelines, applies rigorous methodological assessment using the STROBE checklist to provide sport-specific insights that can guide targeted prevention strategies for volleyball players.

## 2 Methods

This review was conducted following the PRISMA (Preferred Reporting Items for Systematic Reviews and Meta-Analyses) 2020 guidelines for the conduct and reporting of systematic reviews, and the PRISMA-S extension (Preferred Reporting Items for Systematic Reviews and Meta-Analyses Search Extension) was additionally used to ensure full transparency of the search strategy (10). The study was registered in the International Prospective Register of Systematic Reviews (PROSPERO) under ID CRD42024510881.

### 2.1 Search strategy

A comprehensive search was performed in three electronic databases—PubMed, Web of Science, and SCOPUS—from inception to February 21, 2024. The following search terms were used:

“((volleyball players) OR (“volleyball athletes”) OR (volley)) AND ((injury) OR (injur*) OR (injuries) OR (prevalence) OR (incidence)) AND ((ACL) OR (“Anterior Cruciate Ligament”))”.

Two reviewers (LBS and ACM) independently screened titles, abstracts, and full texts. Disagreements were resolved by a third reviewer (TTS). An updated search was conducted on October 24, 2025, using the same criteria, but no additional eligible studies were identified.

### 2.2 Eligibility criteria

Eligibility was defined using the PECOS framework (Population, Exposure, Comparison, Outcome, Study design), as summarized in Table 1. Only peer-reviewed articles published in English were considered eligible.

**Table 1:** PECOS eligibility criteria.

Domain	Inclusion criteria	Exclusion criteria
Population	Volleyball athletes of any age or sex, including injured and non-injured players	Non-volleyball athletes and/or individuals without ACL injuries
Exposure	Participation in volleyball training and/or match activities, as defined by each study (e.g., athlete-exposures, hours of exposure, matches, or training sessions)	Other sports
Comparison	Differences across sex, age groups, playing positions, or competition levels when reported	–
Outcome	ACL injury incidence (injuries per exposure unit) or prevalence (proportion of athletes with ACL injury)	Studies that did not report ACL injury incidence or prevalence in volleyball athletes
Study design	Prospective or retrospective cohort studies reporting epidemiological data on ACL injuries in volleyball.	Systematic reviews, meta-analyses, case studies, letters, clinical trials, commentaries, and narrative reviews

### 2.3 Data extraction

Data were independently extracted by two reviewers using a standardized spreadsheet. Extracted variables included:

- Author and year
- Study design and duration
- Sample size and demographics
- Level of play and playing position
- ACL injury incidence and/or prevalence
- Mechanism of injury
- Type of exposure (training or competition)
- Injury definitions
- Post-injury management (if reported)

Disagreements were resolved by a third reviewer. The definitions of “incidence” and “prevalence” were preserved as reported in each study.

Due to heterogeneity in study designs, populations, and outcome definitions, meta-analysis was not conducted, and findings were synthesized narratively.

#### 2.3.1 Prevalence estimation

For studies that did not report prevalence directly but provided the number of ACL injuries and the total sample size, prevalence was estimated using the formula:

$$\mathrm{Prevalence}(\text{\%})=\left(\frac{\mathrm{number}\;\mathrm{of}\;\mathrm{ACL}\;\mathrm{injury}\;\mathrm{cases}}{\mathrm{total}\;\;\mathrm{sample}}\right)X100$$

### 2.4 Quality assessment

The STROBE (Strengthening the Reporting of Observational Studies in Epidemiology) checklist was used to evaluate the clarity and completeness of reporting in each included study (11). This tool does not directly assess risk of bias but offers insights into methodological transparency. Each of the 33 checklist items was evaluated and scored as 1 (clearly reported) or 0 (not reported or unclear), following commonly used adaptations of STROBE for observational epidemiological reviews. Scoring was performed independently by two reviewers. Discrepancies in scoring were resolved through consensus. To ensure greater transparency in grading, studies were categorized as high (≥25 points), moderate (15–24 points), or low (<15 points) reporting quality based on total STROBE scores. These thresholds are consistent with previous applications of modified STROBE scoring systems and facilitate clearer interpretation of reporting completeness across the included studies.

## 3 Results

### 3.1 Study selection

**Figure 1:**
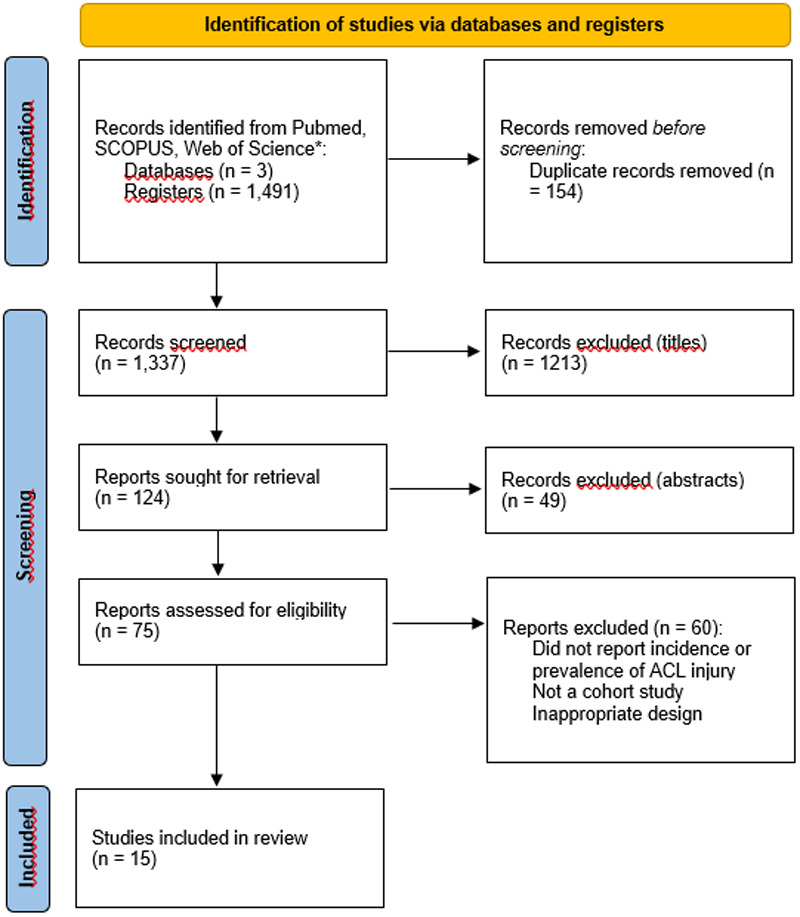
PRISMA flow diagram showing the identification, screening, eligibility assessment, and inclusion of studies in the systematic review.

A total of 1,491 records were retrieved from the databases. After removing duplicates, 1,337 titles remained. Following title and abstract screening, 124 articles were reviewed in full, and 15 studies met all inclusion criteria (Figure 1).

### 3.2 Methodological quality

The reporting quality of the included studies, assessed using the STROBE checklist, ranged from 10 to 31 out of 33, with an average score of 21 points, indicating moderate overall quality. Most studies adequately reported basic elements such as study design, objectives, settings, and participant characteristics (Items 1a, 2–5, and 14a). In contrast, several methodological domains were frequently underreported.

Common weaknesses included lack of detail on sample size calculation (Item 10), handling of missing data (Item 12c), and sensitivity analyses (Item 12e)—items that were either omitted or poorly described in the majority of studies. Additionally, confounding control methods (Items 12a and 16a) and discussion of generalizability (Item 21) were inconsistently addressed. Only a few studies provided full statistical detail, including subgroup or interaction analyses (Item 12b), and even fewer discussed the role of funding sources (Item 22).

To improve transparency, representative examples of reporting limitations were added. For instance, some studies did not specify whether exposure referred to athlete-hours or athlete-exposures, while others did not clearly describe ACL diagnostic confirmation methods.

The focus on reporting trends across all studies replaces previous emphasis on the highest and lowest individual STROBE scores, providing a more balanced synthesis of methodological quality.

Supplementary Table S1 provides detailed item-level STROBE scores for each included study.

### 3.3 Study characteristics and demographics

The 15 included studies represented 3,313,248 volleyball athletes, aged between 10 and 70 years, with a mean age around 20 years. Nine studies included only female athletes, while six included both sexes. Sample sizes ranged from 11 to over 1.7 million athletes.

Most studies employed prospective or retrospective cohort designs, although several described their approach as descriptive epidemiology. The duration of data collection ranged from 1 to 16 years. ACL injury data were reported either as incidence rates, prevalence estimates, or both (Table 2).

**Table 2:** Characteristics of included studies: study design, sample, exposure time, and ACL injury rates.

Author (year)	Study design/duration	Sample size (F/M)	Age (mean ± SD)	Incidence	Prevalence	ACL injuries (*n*)
Nicolini et al. (2014) (4)	Cross-sectional cohort/1 year	26 (F/M)	Mean 27 years	–	26.9%	7
Joseph et al. (2013) (12)	Descriptive epidemiological/–	Not reported (F)	High school	0.053 per 1,000 AEs (competition); 0.0009 per 1,000 AEs (training); 0.024 per 1,000 AEs (total)	–	20
Beynnon et al. (2014) (13)	Descriptive epidemiological/4 years	Not reported (F)	University	0.447 per 1,000 person-days; adjusted 0.569 per 1,000 person-days	–	1
Patterson et al. (2021) (14)	Prospective cohort/–	130 (F)	19.31 ± 1.1 years	–	0.7%	1
Swenson et al. (2013) (15)	Descriptive epidemiological/6 years	Not reported (F/M)	–	0.00 (M); 0.028 (F) per 1,000 AEs	–	–
Devetag et al. (2018) (16)	Retrospective cohort/5 years	1,488 (F)	16–35 years	–	2.2%	34
Dragoo et al. (2011) (17)	Prospective cohort/1 year	21 (F)	19.5 ± 1.6 years	–	0%	0
Reeser et al. (2015) (18)	Descriptive epidemiological/4 years	Not reported (F)	High school and university	–	–	High school: 8; College: 6
Agel et al. (2007) (19)	Descriptive epidemiological/16 years	Not reported (F)	University	0.178 per 1,000 AEs (training/competition)	–	127
Agel et al. (2016) (20)	Descriptive epidemiological/9 years	Not reported (F)	University	0.06 per 1,000 AEs (95% CI: 0.04–0.08)	–	30
De Loës et al. (2000) (21)	Descriptive epidemiological/7 years	Not reported (F/M)	14–20 years	*F* = 0.004; *M* = 0.002 per 1,000 sport exposure hours	–	F: 14; M: 2
Majewski et al. (2006) (22)	Descriptive epidemiological/10 years	Not reported (F/M)	>10 to >70 years	–	–	47
Vauhnik et al. (2011) (23)	Prospective cohort/1 year	286 (F)	18.1 ± 3.3 years	0.019 per 1,000 h (95% CI: 0.00–0.04)	1%	3
Takahashi and Okuwaki (2017) (24)	Retrospective cross-sectional cohort/9 years	JHSM: 537,640; HSM: 400,448; JHSF: 1,733,960; HSF: 639,238 (athlete-years)	12–18 years	JHSM: 0.08; HSM: 0.48; JHSF: 0.53; HSF: 3.42 (per 1,000 athlete-years; 95% CI provided)	–	–
Mountcastle et al. (2007) (25)	Prospective cohort/9 years	Not reported (F/M)	University	–	–	F: 2; M: 2

AEs, athletic exposures; F, female; M, male; JHSM, junior high school males; HSM, high school males; JHSF, junior high school females; HSF, high school females.

### 3.4 ACL injury incidence and prevalence

Injury rates varied substantially across the included studies, with noticeable differences in how exposure and incidence were calculated. In high school athletes, for example, Joseph et al. (12) reported an incidence of 0.024 per 1,000 athletic exposures, while Beynnon et al. (13), analyzing university-level cohorts, found a higher incidence of 0.569 per 1,000 person-days. In female volleyball players, Vauhnik et al. (23) documented an incidence of 0.019 per 1,000 h of exposure. Reported prevalence values also ranged widely, from 2.2% to 26.9%, depending on sample characteristics, injury definitions, and follow-up periods (4, 16). This variability reflects differences across studies regarding exposure definitions, diagnostic criteria (clinical vs. imaging-confirmed), and participant characteristics.

A summary of ACL injury incidence and prevalence rates across all studies is presented in Table 3.

**Table 3:** Injury mechanisms and ACL injury definitions in included studies.

Authors (year)	Injury mechanisms associated	ACL injury definition
Nicolini et al. (2014) (4)	Female sex (+)	Participants with a clinically diagnosed knee injury, caused by or symptomatic during sports practice, which prevented the athlete from training for a variable period.
Joseph et al. (2013) (12)	Contact injury (16%)	Injury had to occur during an organized practice or competition, require attention from an athletic trainer or physician, and result in restriction from practice or competition for at least 1 day.
	Non-contact injury (31%)	
	Player-surface contact (52%) (+)	
	Injury during training (*n* = 5)	
	Injury during competition (*n* = 15) (+)	
Beynnon et al. (2014) (13)	–	Grade 3 complete ACL rupture in individuals with no prior ACL injury in either leg, occurring during organized university or elite-level events, without direct contact to the knee by external forces (e.g., other athletes, equipment, or the ground).
Patterson et al. (2021) (14)	Non-contact injury (+)	Any injury to the lumbar region or lower limbs sustained during sports participation (training or competition) that prevented the athlete from completing the activity or from participating in the next session.
Swenson et al. (2013) (15)	Female sex (+)	A reportable injury met all of the following: (1) occurred during training or competition; (2) required medical attention by a trainer or physician; (3) resulted in time-loss of ≥1 day.
	Injury during competition (+)	
	Non-contact injury (+)	
Devetag et al. (2018) (16)	Non-contact (97%) (+)	–
	Contact (3%)	
	Landing after attack (62%) (+)	
	General landing (26%)	
	Landing after block (9%)	
	Apparent contact (3%)	
	Outside hitter (42%) (+)	
	Middle blocker (30%)	
	Libero (9%)	
	Setter (18%)	
	Opposite (3%)	
Dragoo et al. (2011) (17)	–	Suspected ACL ruptures were clinically assessed by an orthopedic surgeon with sports medicine training and confirmed via MRI.
Reeser et al. (2015) (18)	High school: 9.3% (+) vs. College: 4.4%	Any condition resulting in the loss of at least 1 day of practice or competition.
Agel et al. (2007) (19)	–	Defined as any injury occurring during intercollegiate training or competition that required medical attention from a certified athletic trainer or team physician and restricted participation for at least one subsequent calendar day.
Agel et al. (2016) (20)	Competition-related injury (+)	Any ACL injury, regardless of contact mechanism. Only new injuries were counted; recurrences were excluded. Diagnosis was confirmed by the initial reporting provider and/or imaging.
	Training-related injury	
De Loës et al. (2000) (21)	–	–
Majewski et al. (2006) (22)	–	ACL injury diagnosis involved radiographic imaging (AP and lateral views) to assess

(+) indicates a higher observed injury rate associated with the variable.

### 3.5 Main risk patterns identified

A consistent pattern across studies was the predominance of non-contact mechanisms as the primary cause of ACL injuries, with most injuries occurring during landing, blocking, or spike-approach actions. For instance, Devetag et al. (16) reported that 97% of ACL injuries occurred via non-contact mechanisms, with 62% happening specifically during landing after an attack. Across studies reporting sex differences, female athletes, particularly adolescents, showed higher ACL injury rates compared with male players. Joseph et al. (12) found that female volleyball athletes had significantly higher ACL injury rates compared to their male counterparts. Reeser et al. (18) observed that high school athletes presented a higher injury prevalence (9.3%) than collegiate players (4.4%), reinforcing the vulnerability of younger athletes engaged in competitive settings.

Notably, post-injury management was insufficiently reported: only two studies described treatment approaches, both indicating that between 75% and 100% of injured athletes underwent surgical reconstruction (12, 16).

## 4 Discussion

### 4.1 Epidemiology of ACL injuries in volleyball

This review revealed that ACL injuries in volleyball athletes are influenced by athlete sex, level of play, exposure duration, and injury mechanisms. In the context of volleyball, where spike landings are a common injury scenario, these distinctions may help contextualize the injury scenarios commonly observed in volleyball. These findings align with previous research indicating that the majority of ACL injuries in volleyball occur during landing, where the knee is subjected to high shear forces and rotational stress (26). The consistent observation that adolescent female athletes, especially those playing in the outside hitter position, are at the greatest risk is supported by extensive literature on biomechanical and neuromuscular factors that heighten ACL strain in this demographic (12, 16, 27). As the included studies were observational, the patterns reported describe associations rather than causal risk factors.

Although these mechanisms were not directly evaluated in the included studies, they help contextualize the epidemiological patterns observed in volleyball athletes. The anterior cruciate ligament (ACL) injuries represent a significant concern across various age groups, particularly among adolescents and younger athletes. Studies indicate that high school athletes often experience higher rates of ACL injuries compared to their collegiate counterparts, potentially due to incomplete neuromuscular maturation, lower technical skill acquisition, and increased hormonal variability in female athletes (3, 5, 28, 29). Moreover, age-related differences in biomechanical patterns have been described in younger athletes (30), which may influence landing control and injury patterns. In contrast, older athletes may be more prone to cumulative joint stress or degenerative changes (31).

In the context of volleyball, where spike landings are a common injury scenario, these distinctions may be especially relevant. Thus, future studies should stratify risk factors and injury mechanisms by age group to clarify whether prevention programs should be tailored based on developmental and physiological differences (32, 33). Across the included studies, non-contact mechanisms accounted for approximately 85%–97% of ACL injuries, and when both sexes were reported, female athletes exhibited 2–4 times higher injury rates than their male counterparts.

Differences in strength and conditioning practices may also account for variations in ACL injury prevalence observed across studies. Collegiate volleyball athletes are generally exposed to structured pre-season and in-season conditioning programs that emphasize neuromuscular control, landing technique, and lower-limb strength, whereas high-school athletes often have limited access to specialized training and preventive programs. This discrepancy may partly explain the higher incidence of ACL injuries among younger players. Moreover, the studies included in this review were conducted in different countries, such as the United States, Japan, Slovenia, and Italy, where variations in training methodologies, coaching resources, and injury surveillance systems could influence reported prevalence rates. Therefore, contextual differences in conditioning and prevention practices should be considered when interpreting epidemiological data across populations. By focusing exclusively on volleyball athletes, this review offers sport-specific insights that complement prior broader reviews on ACL injuries. Unlike reviews that aggregate data from multiple sports or rely on narrative synthesis, the current study systematically analyzes epidemiological patterns specific to volleyball—such as spike landings, positional risks, and age-related trends—thereby providing a refined understanding that supports the development of targeted prevention strategies tailored to the sport's unique biomechanical demands.

### 4.2 Why a volleyball-specific review is needed

#### 4.2.1 Unique biomechanical demands of volleyball

These contextual findings are drawn from broader literature and help interpret the epidemiological patterns observed. Volleyball is a highly dynamic sport characterized by the sequenced execution of vertical jumps, spike landings, and lateral movements, which raises the risk of anterior cruciate ligament (ACL) injuries. These injuries are particularly prevalent among outside hitters who frequently engage in high-impact landings. The unique biomechanical demands of volleyball, especially during spike landings, create distinct challenges compared to sports such as soccer or basketball, where cutting and pivoting are typically implicated in ACL injuries.

In the context of volleyball, studies indicate that ACL injuries primarily arise from single-leg landings under time constraints, often coupled with torso rotation to position for a subsequent play (9, 34). Recent research suggests that the load distribution during these landings can exacerbate the risk of injury. In approach-jump spikes, the penultimate step increases braking demands while trunk rotation and arm swing modulate frontal and transverse plane knee moments, narrowing the motor-control time window after initial contact (≈30–50 ms) and amplifying error sensitivity in single-leg landings (34, 35). These constraints help explain why small deviations in hip–knee–ankle alignment or delayed hamstring/gluteal activation can disproportionately increase ACL load during volleyball-specific tasks.

Similarly, Xu et al. (34) demonstrated that single-leg landings following a spike may elevate the risk of ACL injuries compared to bilateral landings, as these tend to increase lateral shearing forces at the knee joint. Borzucka et al. (36) emphasized that the necessity for exceptional postural control during rapid movements can lead to a destabilization–recovery sequence that further predisposes players to injury.

From a mechanical perspective, vertical ground reaction forces (vGRF) recorded during volleyball landings play a critical role in understanding injury patterns (35) demonstrated that vGRF is a vital kinetic parameter for monitoring potential injuries, and its analysis can inform both developmental and rehabilitative strategies. Moreover, Xu et al. (34) proposed that proper landing strategies—such as adjusting limb dynamics and encouraging bilateral landings—can significantly reduce ACL injury incidence.

These biomechanical considerations differ markedly from other sports. In soccer and basketball, ACL injuries often occur during premeditated cutting or pivoting maneuvers. In contrast, volleyball players are exposed to high-frequency and asymmetrical force adaptations during spontaneous spike landings (34). Literature advocates for tailored training interventions that improve landing mechanics and postural stability. Notably, the 30–50 ms following ground contact have been identified as a critical window in ACL injury occurrence (34).

#### 4.2.2 Limitations of multi-sport reviews

The epidemiology and biomechanics of anterior cruciate ligament (ACL) injuries vary substantially across different sports, reinforcing the need for sport-specific injury prevention strategies (8). The incidence and mechanisms of ACL injuries are closely linked to sport-specific demands. For instance, high school boys participating in football exhibit ACL injury rates nearly four times higher than those in other sports (12). Among female athletes, the risk is also highly variable: ACL injuries are nearly four times more likely to occur in soccer and basketball than in volleyball or softball (12).

Moreover, each sport presents distinct injury mechanisms. In court sports like basketball, non-contact mechanisms—such as jump landings or cutting maneuvers—are predominant. In contrast, soccer-related ACL injuries often involve contact events, accounting for 55%–80% of cases (37). The biomechanical demands further diverge: while soccer is characterized by rapid directional changes and extended running phases, volleyball is centered on vertical explosiveness, positional jumping, and high-frequency landing tasks with fewer multidirectional accelerations (38). These sport-specific discrepancies limit transferability from cross-sport reviews: exposure denominators (athletic exposures vs. player-hours vs. athlete-years), injury case definitions (first-time vs. recurrent; contact vs. non-contact), and task demands (cutting/pivoting vs. vertical, asymmetrical landings) differ, biasing pooled estimates and blurring practical guidance for volleyball.

These differences have important implications for injury prevention. Although neuromuscular training programs have shown strong efficacy across high-risk populations (39, 40), their success is contingent on sport-specific adaptation. Programs that focus on balance, agility, and landing mechanics have demonstrated significant reductions in ACL injury rates, particularly among female athletes in jumping sports (41, 42).

Importantly, athletes' awareness and knowledge of ACL injury risks also vary by sport. Alsaeed et al. (43) reported that athletes in high-contact sports display greater awareness of ACL injury mechanisms compared to those in lower-risk environments. This reinforces the need for not only physical prevention strategies but also targeted educational initiatives aligned with the demands and risks of each sport.

#### 4.2.3 Need for sport-specific prevention strategies

In the context of injury prevention strategies for volleyball players, it is essential to consider the sport's distinct biomechanical and positional demands. Volleyball athletes are especially prone to injuries resulting from repetitive actions such as spike landings, abrupt lateral transitions, and asymmetrical load patterns associated with different playing positions (44, 45). These stressors require the development of prevention programs that are not only sport-specific, but also tailored to individual profiles defined by sex, age, and playing role.

Although general injury prevention protocols such as STOP-X and FIFA 11+ have demonstrated efficacy across various sports (46), their translation to volleyball may be limited without appropriate modifications. Volleyball involves unique demands, including high-frequency jumping and rapid transitions between blocking, setting, and spiking, which are not directly addressed by generic programs. As highlighted by Bahr and Bahr (47), warm-up routines that include volleyball-specific drills emphasizing dynamic movement, proprioceptive control, and jump-landing mechanics are more likely to reduce injury incidence and enhance performance.

Furthermore, certain injuries—such as shoulder pathologies—are prevalent in volleyball due to repetitive overhead actions. Strength training protocols targeting the shoulder girdle have proven effective in improving muscular balance and joint stability, thereby reducing injury prevalence (44, 48, 49). Similarly, proprioceptive-focused neuromuscular training has been associated with reductions in ankle sprain risk, another common injury in volleyball (50, 51).

Importantly, prevention strategies should account for interindividual differences. Stratified approaches based on sex, age, and positional demands allow for more precise intervention. For example, female volleyball players may benefit from programs addressing biomechanical risk factors such as dynamic valgus and core instability, while male players may require adjustments in training loads due to greater jump volume (47, 52). Positional considerations are equally important: outside hitters and middle blockers engage in distinct movement patterns and loading profiles, requiring targeted adaptations in training content and monitoring.

#### 4.2.4 Comparative perspective: volleyball versus basketball and soccer

Anterior cruciate ligament (ACL) injuries present a significant concern in sports such as basketball, soccer, and volleyball. While these sports share some biomechanical elements, their injury mechanisms, incidence rates, and optimal prevention strategies differ substantially, underscoring the importance of sport-specific approaches.

In soccer, ACL injuries often result from high-velocity cutting and pivoting maneuvers, commonly exacerbated by contact with other players. Notably, however, many injuries in female soccer athletes still occur through non-contact mechanisms—(53) reported that approximately 88% of ACL injuries in female soccer players happened without direct knee contact. Moreover, the overall incidence of ACL injuries in soccer is higher than in sports such as field hockey or gymnastics (54), reinforcing the need for targeted biomechanical optimization and prevention protocols.

Basketball also poses a high risk for ACL injuries, especially among female athletes. These injuries frequently occur during abrupt landings and changes in direction, often through non-contact mechanisms (5, 55). Neuromuscular training interventions—focused on landing control and body awareness—have proven effective in reducing ACL injury incidence in this context (5, 40).

Volleyball, by contrast, is considered lower risk than soccer or basketball but still features meaningful non-contact ACL injury mechanisms. Injuries most commonly arise during spike landings and blocking actions—movements marked by single-leg landings and minimal contact with opponents (34, 56). These landings impose asymmetric loads on the knee joint, a risk that is amplified when proper mechanics are not maintained (57). In contrast to the more multidirectional, horizontal decelerations seen in soccer and basketball, volleyball is typified by repetitive vertical propulsion and recovery.

Furthermore, while preventive programs such as FIFA 11+ or STOP-X have shown efficacy in soccer and basketball settings, their direct application to volleyball may be limited. These frameworks often fail to account for the sport's distinct biomechanical patterns, particularly asymmetrical jump-landing tasks (58). Systematic reviews suggest that volleyball-specific modifications are needed to address injury risks effectively (59).

Comparative epidemiology also reveals notable sex-based and sport-based differences. Female basketball players face ACL injury risks nearly four times greater than female volleyball players (3, 60). This heightened risk is associated with factors such as neuromuscular control challenges and valgus collapse during pivoting—a movement less typical in volleyball (61). In contrast, volleyball's primary mechanism of injury—single-leg landings post-spike—demands an alternative preventive focus.

Fatigue is an additional factor that influences injury risk, particularly in volleyball. Repetitive landing tasks lead to fatigue-induced biomechanical alterations that can compromise joint stability and increase injury likelihood (62). Prevention strategies in volleyball must, therefore, integrate load management alongside technique refinement.

While some injury mechanisms may overlap across team sports, volleyball's unique biomechanics, lower player-to-player contact, and emphasis on vertical asymmetric landings necessitate tailored approaches. The application of generalized injury prevention strategies developed for basketball or soccer—without adjustments—may overlook key volleyball-specific risk factors. Customized interventions based on these distinct movement patterns are critical for effectively reducing ACL injury risk in volleyball athletes.

### 4.3 Influence of sex on ACL injury risk

Sex-related risk in volleyball is best understood through interacting anatomical and neuromuscular factors that shape knee loading during landings, rather than through incidence figures alone. Anatomical differences, such as a wider Q-angle, increased knee valgus, greater knee joint laxity, and hormonal fluctuations throughout the menstrual cycle, contribute to heightened susceptibility among female athletes (63–65). These factors are further exacerbated in sports like volleyball, where frequent jumping and landing place repetitive strain on the lower extremities, increasing the risk of non-contact ACL injuries. Specifically, the landing mechanics of female athletes often exhibit greater knee valgus, reduced hip flexion, and diminished hamstring activation—each of which is strongly associated with increased ACL loading (9, 33).

### 4.4 Role of biomechanics and neuromuscular factors

Biomechanical analyses have demonstrated that during landing, female athletes are more likely to exhibit an inward collapse of the knee (dynamic knee valgus), which is a critical mechanism for ACL injury. Such movement patterns are influenced not only by anatomical factors but also by neuromuscular deficits, including delayed activation of protective muscles such as the hamstrings and gluteals (64, 66). The repetitive nature of jump landings—especially among outside hitters who frequently engage in powerful, high-risk spike attacks—increases the cumulative load on the knee joint (3, 40).

Although general prevention programs such as STOP-X have proven effective in reducing dynamic valgus and ACL injury risk across multiple populations (67), the unique biomechanical demands of volleyball necessitate complementary sport-specific adaptations. Volleyball players—particularly outside hitters—frequently perform high-frequency jumping and directional changes, which impose complex loading patterns on the knee. Research has shown that during forward and lateral landings, knee valgus angles in volleyball players can reach 5.8° and 8.8°, respectively, increasing ACL strain (68).

Given these movement patterns, incorporating drills that simulate spike landings and lateral transitions may enhance the effectiveness of generic prevention strategies. Neuromuscular warm-up routines, as advocated by DiStefano et al. (69) have been effective in reducing lower extremity injuries in youth athletes, including ACL tears (70) demonstrated that experienced volleyball athletes adopt safer landing kinematics compared to novices, emphasizing the importance of skill-specific motor training. Similarly, Fatahi et al. (71) and Yang et al. (72) have highlighted the critical role of knee and ankle joint mechanics in mitigating non-contact ACL injury risk, particularly during tasks such as stop-jumps and side-cutting maneuvers.

Tarantino (9) underscored the relevance of neuromuscular control in both injury prevention and rehabilitation for female volleyball players. Consequently, mechanisms in volleyball hinge on frontal/transverse plane control and timely hamstring–gluteal activation within the first 30–50 ms of stance; interventions that progressively shift from bilateral to unilateral landings and increase task complexity align more closely with these sport-specific demands.

### 4.5 Methodological influences on reported incidence

The incidence of ACL injuries in volleyball athletes is significantly influenced by various methodological factors, including definitions of injury, exposure measurements, and participant characteristics. Comparability is further limited by inconsistent handling of multiple events per athlete, varying inclusion of practice vs. match exposures, and incomplete reporting of denominators, which can inflate or deflate incidence estimates independent of true risk. This underscores the pressing necessity for targeted, evidence-based prevention programs within the sport (63, 73). The documented elevation in ACL injury rates among female athletes can be attributed to various intrinsic risk factors, including altered biomechanical profiles such as dynamic knee valgus and decreased core stability, which particularly impact high school athletes (74, 75).

### 4.6 Effectiveness of neuromuscular training for ACL prevention

Neuromuscular training has been highlighted as an effective preventive measure against ACL injuries. Myer et al. (65) confirmed that age-disparate responses to neuromuscular training could lead to marked reductions in injury incidence, suggesting that earlier intervention may yield better outcomes in at-risk populations. The importance of integrating structured neuromuscular training protocols has been substantiated by numerous studies indicating that such interventions significantly reduce injury risk by modifying poor movement patterns associated with ACL injuries (40, 66). For instance, programs that enhance core strength and improve landing technique have been shown to be beneficial (76, 77).

In support of these findings, a comprehensive summary of systematic reviews by Stephenson et al. (78) identified neuromuscular training as one of the most effective and consistently supported strategies for sports injury prevention, particularly for ACL injuries. Their umbrella review emphasized that programs combining plyometrics, balance, strength, and technique training yield significant reductions in injury risk across various athletic populations. However, they also noted that sport-specific adaptations enhance adherence and effectiveness. Therefore, integrating volleyball-specific elements into neuromuscular protocols—such as spike-landing simulations and lateral cutting drills—may further amplify injury prevention outcomes in this population.

### 4.7 Practical recommendations for coaches and clinicians

Given the evidence linking neuromuscular training and improved biomechanical stability during sport-specific movements, coaches, physiotherapists, and strength and conditioning professionals should prioritize incorporating ACL injury prevention exercises early in athlete development (79, 80). This emphasis on preventative training is echoed in a comprehensive review by Webster and Hewett (81), which found that well-structured training programs can lead to a significant reduction in both all and non-contact ACL injuries among athletes. In light of this evidence, early adoption of these training protocols is recommended to foster better performance outcomes while mitigating injury risks, particularly in high-risk groups such as female volleyball athletes (82).

### 4.8 Position-specific risk: outside hitters

The influence of player position is a critical factor in understanding ACL injury risk. Outside hitters, who are responsible for executing high-velocity spike landings, are exposed to greater vertical ground reaction forces, making them more vulnerable to ACL injuries (12, 16). This aligns with the concept of “functional biomechanics,” where the specific movements required by each position can create unique injury risks (83, 84). The frequent lateral movements, sudden directional changes, and landing dynamics inherent to the outside hitter role compound the risk, especially among younger athletes who may lack the neuromuscular control to safely absorb impact forces.

### 4.9 Integrating sport-specific prevention strategies

The documented high incidence of ACL injuries in volleyball, particularly among young female athletes, underscores the need for sport-specific preventive measures that are grounded in robust theoretical frameworks and practical training applications. Such measures should include:

- Regular neuromuscular training that enhances core stability and improves landing mechanics (3, 40).
- Position-specific drills that replicate the high-risk movements encountered by outside hitters.
- Educational sessions for coaches and athletes focusing on safe landing techniques and dynamic balance control.

### 4.10 Limitations and future directions

While this review provides a comprehensive overview of ACL injury patterns in volleyball, several limitations must be acknowledged. First, the heterogeneity in study designs, injury definitions, and population characteristics may have influenced the reported incidence rates and hindered direct comparisons across studies. Additionally, most of the included studies were conducted in high-income countries, which limits the generalizability of findings to broader and more diverse athletic populations. Another important limitation is the inability to determine clear temporal trends in ACL injury rates among volleyball players. Although some data, such as those from the NCAA, suggest a possible decline in collegiate sports, this trend could not be confirmed in the present review due to inconsistent reporting formats and the lack of longitudinal studies specifically focused on volleyball. The included studies span over two decades, but no consistent pattern, whether increasing, decreasing, or stable, was observed. This is primarily due to substantial heterogeneity in how incidence is reported (e.g., per 1,000 athletic exposures, hours, person-days, or athlete-years), as well as differences in sample characteristics and injury definitions. As such, caution is warranted when interpreting or comparing rates over time. Therefore, future research should prioritize the standardization of injury surveillance methods, cross-cultural epidemiological comparisons, and long-term monitoring of injury patterns. Moreover, the development and evaluation of sport-specific prevention strategies tailored to the unique demands of volleyball remain essential to effectively reduce the burden of ACL injuries in this population.

### 4.11 Implications for injury prevention in volleyball

The findings of this systematic review reveal that adolescent female volleyball athletes, particularly those playing as outside hitters, are at the highest risk for anterior cruciate ligament (ACL) injuries. This heightened vulnerability among young female players may be attributed to several factors, including hormonal influences, biomechanical differences, and specific demands of the outside hitter position, which often involves high-frequency jumping and landing tasks. Given the predominance of non-contact mechanisms in ACL injuries, particularly during spike landings in competitive settings, targeted prevention strategies become essential.

Effective prevention programs should prioritize neuromuscular training and landing technique education, with a focus on enhancing lower limb strength, dynamic stability, and proprioception. Such programs should be introduced early in the athlete's development, providing young players with the foundational skills to execute safe and controlled landings. Regular assessment of landing mechanics through video analysis and on-court feedback can further reinforce proper techniques and reduce injury risk.

Moreover, the implementation of sport-specific injury surveillance and intervention programs is crucial to monitor injury trends and evaluate the effectiveness of preventive strategies in real-world settings. These programs should be tailored to the specific demands of volleyball, considering the unique movement patterns, positional roles, and competition levels. By systematically identifying high-risk athletes and modifying training practices accordingly, the overall burden of ACL injuries in volleyball can be effectively reduced, enhancing athlete safety and prolonging athletic careers. Based on existing evidence, neuromuscular training programs should include 15–20 min sessions performed 2–3 times per week, integrating balance, plyometric, and core-stabilization exercises with progressive difficulty. Drills should evolve from bilateral to unilateral landings and from stable to unstable surfaces to optimize transfer to sport-specific tasks.

## 5 Conclusions

ACL injuries remain a significant concern in sports, with volleyball presenting unique injury mechanisms compared to soccer and basketball. While all three sports share some biomechanical elements—such as jump-landing tasks—volleyball is distinguished by high-frequency vertical jumps, single-leg landings, and minimal contact scenarios. These factors contribute to a distinct risk profile that is not adequately addressed by prevention programs developed for other sports.

The current review highlights the urgent need for volleyball-specific injury prevention strategies. This includes tailoring neuromuscular training to the sport's unique movement patterns, incorporating fatigue management, and accounting for positional and sex-based risk factors. Comparative evidence underscores that applying generalized prevention protocols across sports may obscure key sport-specific demands, ultimately reducing their effectiveness.

Future research should prioritize longitudinal studies and randomized controlled trials focusing exclusively on volleyball populations. These efforts will support the development of targeted, evidence-based interventions capable of mitigating ACL injury risks in volleyball athletes and enhancing long-term athletic health.
