# Supplementary material for: Understanding ACL injuries in volleyball: a systematic review of epidemiology and risk factors
Source: Front Sports Act Living. 2025 Dec 8;7:1675136. doi: 10.3389/fspor.2025.1675136 (PMC12719452; doi:10.3389/fspor.2025.1675136)
Supplement: Supplementary file 1 [file Table1.docx]

**Supplementary Table 1. STROBE Checklist Scores of Included Studies**

| **Author (Year)** | **1a** | **1b** | **2** | **3** | **4** | **5** | **6a** | **6b** | **7** | **8** | **9** | **10** | **11** | **12a** | **12b** | **12c** | **12d** | **12e** | **13a** | **13b** | **13c** | **14a** | **14b** | **14c** | **15** | **16a** | **16b** | **16c** | **17** | **18** | **19** | **20** | **21** | **22** | **TOTAL** |
| --- | --- | --- | --- | --- | --- | --- | --- | --- | --- | --- | --- | --- | --- | --- | --- | --- | --- | --- | --- | --- | --- | --- | --- | --- | --- | --- | --- | --- | --- | --- | --- | --- | --- | --- | --- |
| Nicolini et al. (2014) | x | x | x | x | - | x | x | x | x | x | - | x | x | x | - | - | - | - | x | - | - | x | - | x | x | - | - | - | x | x | x | x | - | x | 21 |
| Joseph et al. (2013) | x | x | x | - | x | x | x | x | x | x | - | x | x | x | x | x | x | x | - | - | - | - | - | x | x | x | - | - | x | x | x | x | x | - | 24 |
| Beynnon et al. (2014) | x | x | x | x | x | x | - | - | x | x | x | - | x | x | x | - | - | - | - | - | - | x | - | x | x | x | - | - | x | x | x | x | x | x | 22 |
| Patterson et al. (2021) | x | x | x | x | - | - | x | x | x | x | - | x | x | x | x | - | - | - | x | x | - | x | - | - | - | x | x | - | x | x | x | x | x | x | 23 |
| Swenson et al. (2013) | - | x | x | x | x | x | x | - | x | x | - | x | x | x | x | x | - | - | - | - | - | - | - | x | x | x | - | - | - | x | x | x | x | x | 21 |
| Devetag et al. (2018) | x | x | x | - | x | x | x | - | x | x | - | x | x | - | - | - | - | - | x | - | - | x | - | x | x | - | - | - | x | x | - | x | - | - | 17 |
| Dragoo et al. (2011) | x | x | x | x | x | x | x | x | x | x | x | x | x | x | x | x | x | x | x | x | - | x | - | x | x | x | x | - | x | x | x | x | x | x | 31 |
| Reeser et al. (2015) | x | x | x | x | x | x | - | - | - | x | - | - | x | x | - | - | - | - | - | - | - | - | - | - | x | x | - | - | x | x | x | x | x | x | 17 |
| Agel et al. (2007) | x | x | x | - | - | x | x | - | x | x | - | x | x | x | x | x | - | - | - | - | - | - | - | x | x | x | - | - | x | x | - | x | x | - | 19 |
| Agel et al. (2016) | x | x | x | - | x | x | x | - | x | x | - | x | x | x | x | - | - | - | - | - | - | - | - | x | x | x | - | - | x | x | x | x | - | - | 19 |
| Loës et al. (2000) | - | x | x | - | x | x | x | - | x | x | - | x | x | x | x | - | - | - | x | - | - | x | - | x | x | x | - | - | x | x | x | x | x | - | 21 |
| Majewski et al. (2006) | - | x | x | - | - | x | x | - | - | x | - | x | - | - | - | - | - | - | x | - | - | - | - | x | x | - | - | - | x | - | - | - | - | - | 10 |
| Vauhnik et al. (2011) | x | x | x | x | x | x | x | - | x | x | - | x | x | x | x | - | x | x | x | - | x | x | - | x | x | x | - | - | x | x | x | x | x | - | 26 |
| Takahashi & Okuwaki (2017) | x | x | x | - | x | x | x | - | x | x | - | x | x | x | - | - | - | - | x | - | - | x | - | x | x | x | - | - | x | x | x | x | x | x | 22 |
| Mountcastle et al. (2007) | x | x | x | x | x | x | x | - | x | x | - | x | x | x | x | x | - | - | x | - | - | x | - | x | x | x | - | - | x | x | x | x | x | - | 24 |

STROBE Checklist – Item Descriptions: 1a – Clearly indicate the study design using a commonly used term in the title or abstract. 1b – Provide an informative and balanced abstract summarizing what was done and what was found. 2 – Explain the scientific background and rationale for the investigation being reported. 3 – State specific objectives, including any pre-specified hypotheses. 4 – Present key elements of study design early in the paper. 5 – Describe the setting, locations, and relevant dates, including periods of recruitment, exposure, follow-up, and data collection. 6a – Give the eligibility criteria, and the sources and methods of case ascertainment and control selection. Give the rationale for the choice of cases and controls. 6b – For matched studies, give matching criteria and the number of controls per case. 7 – Clearly define all outcomes, exposures, predictors, potential confounders, and effect modifiers. Give diagnostic criteria, if applicable. 8 – For each variable of interest, give sources of data and details of methods of assessment (measurement). Describe comparability of assessment methods if there is more than one group. 9 – Describe any efforts to address potential sources of bias. 10 – Explain how the study size was arrived at. 11 – Explain how quantitative variables were handled in the analyses. If applicable, describe which groupings were chosen and why. 12a – Describe all statistical methods, including those used to control for confounding. 12b – Describe any methods used to examine subgroups and interactions. 12c – Explain how missing data were addressed. 12d – If applicable, explain how matching of cases and controls was addressed. 12e – Describe any sensitivity analyses. 13a – Report numbers of individuals at each stage of study (e.g., numbers potentially eligible, examined for eligibility, confirmed eligible, included in the study, completing follow-up, and analyzed). 13b – Give reasons for non-participation at each stage. 13c – Consider use of a flow diagram. 14a – Give characteristics of study participants (e.g., demographic, clinical, social) and information on exposures and potential confounders. 14b – Indicate number of participants with missing data for each variable of interest. 15 – Report numbers of outcome events or summary measures over time. 16a – Give unadjusted estimates and, if applicable, confounder-adjusted estimates and their precision (e.g., 95% confidence intervals). Make clear which confounders were adjusted for and why they were included. 16b – Report category boundaries when continuous variables were categorized. 16c – If relevant, consider translating estimates of relative risk into absolute risk for a meaningful time period. 17 – Report other analyses done (e.g., analyses of subgroups and interactions, and sensitivity analyses). 18 – Summarize key results with reference to study objectives. 19 – Discuss limitations of the study, considering sources of potential bias or imprecision. Discuss both the direction and magnitude of any potential bias. 20 – Give a cautious overall interpretation of results considering objectives, limitations, multiplicity of analyses, results from similar studies, and other relevant evidence. 21 – Discuss the generalizability (external validity) of the study results. 22 – Give the source of funding and the role of the funders for the present study and, if applicable, for the original study on which the present article is based.
